# The role of AGG interruptions in the *FMR1* gene stability: A survey in ethnic groups with low and high rate of consanguinity

**DOI:** 10.1002/mgg3.946

**Published:** 2019-08-27

**Authors:** Esther Manor, Raphael Gonen, Benjamin Sarussi, Danielle Keidar‐Friedman, Jay Kumar, Hiu‐Tung Tang, Flora Tassone

**Affiliations:** ^1^ Faculty of Health Science, Ben‐Gurion University of the Negev Genetic Institute Soroka University Medical Center Beer Sheva Israel; ^2^ Nuclear Research Center Negev Beer‐Sheva Israel; ^3^ Department of Life Sciences Ben Gurion University Beer Sheva Israel; ^4^ Department of Biochemistry and Molecular Medicine, School of Medicine University of California Davis Sacramento USA; ^5^ MIND Institute, Medical Center University of California Davis Sacramento USA

**Keywords:** AGG, consanguinity, *FMR1*, full mutation, premutation, prevalence

## Abstract

**Background:**

The prevalence and the role of AGG interruptions within the *FMR1* gene in the normal population is unknown. In this study, we investigated the frequent of AGG loss, in one or two alleles within the normal population. The role of AGG in the *FMR1* stability has been assessed by correlating AGG loss to the prevalence of premutation/full mutation in two ethnic groups differing in their consanguinity rate: high versus low consanguinity rate (HCR vs. LCR).

**Methods:**

The CGG repeat allele size and AGG presence were measured in 6,865 and 6,204 females belonging to the LCR (5%) and HCR (>45%) groups, respectively, by Tripled‐Primed‐PCR technique.

**Results:**

A lower prevalence of the premutation was observed in the HCR (1:158) as compared to the LCR group (1:128). No full mutation was found in the HCR females while in the LCR group the prevalence found was 1:1,149. Homozygosity rate was higher in the HCR population compared to the LCR group.The overall AGG loss was higher in the HCR population than in the LCR and increased with increased CGG repeat number in both ethnic groups.

**Conclusions:**

Although we observed a significantly higher rate of homozygosity and AGG loss in the HCR group, this did not affect the prevalence of the premutation and full mutation in this population. Their prevalence was significantly lower than in the LCR population. Finally, we discuss whether the loss of AGG could be also a polymorphic event but not only a stabilizing factor.

## 1 INTRODUCTION

Fragile X syndrome (FXS) is the most common inherited cause of intellectual disabilities that occurs in approximately 1/4,000 males and 1/8,000 females (Crawford, Acuña, & Sherman, 2001; Hunter et al., 2014). The majority of the cases result from a dynamic mutation caused by the amplification of a CGG trinucleotide repeat, greater than 200, within the 5′ untranslated region of the *FMR1* gene located on the long arm of the X chromosome (Kremer et al., 1991; Oberle et al., 1991; Verkerk et al., 1991; Yu et al., 1991). As a result, the expression of the *FMR1* protein, FMRP, an important protein in brain development, is prevented through methylation of the promoter of the *FMR1* gene. Normal and intermediate alleles (6–44 and 45–54 CGG repeats, respectively) have AGG interruptions that usually occur after every 9 or 10 CGG triplets (Yrigollen et al., 2014) Premutation alleles (55–200 CGG repeats), generally have 0 or 1 AGG interruption. It has been demonstrated that, in addition to the CGG repeat number, increased instability correlates with AGG loss (Gunter et al., 1998; Nolin et al., 2015, 2013; Yrigollen et al., 2012, 2014). The loss of AGG repeat occurs at the 3'‐end, creating a long pure (CGG)n stretch with higher mutability (Kunst & Warren, 1994; Limprasert, Thanakitgosate, Jaruthamsophon, & Sripo, 2016).

Since 2010, depending on the CGG repeats length, three major range categories have been used in Israel: a normal range with <57 CGG repeats and almost no risk to *FMR1* expansion, a premutation range with 58–199 CGG repeats and an increased risk for *FMR1* expansion in the following generations. The increased risk of CGG repeat expansion varies from 3% at 59–69 CGG to 69% and 100% at 70–80 and >90 CGG repeats, respectively (Nolin et al., 2003; Yrigollen et al., 2012). Individuals with a premutation allele are at risk of developing two main clinical manifestations: the fragile X‐associated tremor/ataxia syndrome and the fragile X‐associated primary ovarian insufficiency (reviewed by Hall and Berry‐Kravis (2018) and Fink et al. (2018)). The third category is the full mutation with >200 CGG repeats. In this range, clinical involvements are fully expressed in males and less in females due to the presence of the second X chromosome carrying a normal allele. Expansion to a full mutation occurs almost exclusively when a premutation allele is transmitted from mother to child, and only rarely from father to daughter (Alvarez‐Mora et al., 2017; Zeesman et al., 2004).

Eichler et al. (1994) suggested that AGGs interspersed within the *FMR1* repeat region increase its stability. Since then, the importance and significance the AGG interception in the *FMR1* gene has been extensively studied (Nolin et al., 2015, 2013; Yrigollen et al., 2012, 2014).

The concept that AGG interruptions are playing an important role in the *FMR1* allele stability is widely accepted; however, there are evidences that raise some questions. First, allele expansion occurs mainly during maternal but not paternal transmission, regardless AGG loss or CGG repeat length. Second, although *FMR1* allelic mosaicism is generally characterized by the presence of a premutation and a full mutation alelle, it has been reported also within the normal CGG repeat range (3 alleles of different sizes) regardless AGG loss (Sharony et al., 2012; Wakeling, Nahhas, & Feldman, 2014). Indeed, in this study we report cases with AGG loss in both alleles in the normal CGG repeat length which appears to be in contrast with the expectation that the loss of AGG interruptions causing CGG repeat instability.

To date, the mechanism by which instability leads to "mosaicism" is not known. A strong correlation has been found between CGG repeats length and AGG loss only between 58 and up to 90 CGG repeats. Beyond 90 CGG repeats length *FMR1* expand in almost all the cases (Domniz et al., 2018; Nolin et al., 2015, 2013; Yrigollen et al., 2012, 2014).

In this study, we aimed to further explore the role of AGG interruptions in the stability of the *FMR1* gene and perform haplotype analysis using microsatellites located near the *FMR1* gene, to investigate their potential association with the loss of AGG interruptions in two populations. We compared two groups: one of Jewish ethnicity and the other of Bedouin ethnicity, mainly differing in their consanguinity rate. Consanguinity increases the homozygosity and thus potentially should increase the rate of AGG loss in the population. We also investigated how consanguinity may affect the prevalence of *FMR1* premutation/full mutation alleles in the screened population.

The Bedouins group had a high consanguinity rate (HCR) between 45.2% and 70.1% (from the survey of the Israeli Health department of 1,53,500 Arabs, 2010 about consanguineous marriages among the Arab population in Israel (Naamana, Romano Zalica, Kabbah, & Shohat, 2011). The Bedouin‐Arabs, residing mostly in the Negev desert, comprise ~250,000 individuals. Within the Muslims, consanguineous marriages are the most frequent among the Negev Bedouins (Zlotogora, 2014).

The other group included a Jewish community with relatively low consanguinity rate (less than 5%, LCR) as it was constituted by immigrants among whom the marriages were random. To further study the role of the AGG interruptions in the *FMR1* stability, we compared CGG repeats length, AGG loss patterns and homozygosity rate, in each CGG repeats length category, in the two ethnic groups.

## 2 MATERIALS AND METHODS

### 2.1 Subjects

More than 850,000 habitants are living in the Negev region, among them, are the Bedouins which are represented by approximately 250,000 habitants (Zlotogora, 2014). Since 2010 Fragile X DNA testing is offered free of charge for all the ethnic groups in Israel, as the Ministry of Health covers the cost of the diagnostic test.

Between 2011 and 2017, a total of 17,087 females were admitted to the Human Genetic Laboratory of the Soroka University, Medical Center, which serves the entire Negev region in Israel and their CGG repeat allele size was determined. A total of 9,194 females were of Jewish ethnicity and 7,893 females were of Bedouin ethnicity. The Bedouin and the Jewish ethnic groups were defined as: High Consanguinity Rate (HCR, ~45%–70% consanguinity) and Low Rate Consanguinity (LCR, ~5% consanguinity), respectively. The pattern of AGG loss was assessed in 12,769 (6,204 HRC and 6,565 LRC females) of the 17,087 tested females.

We also measured the CGG repeat allele size in 323 males that were admitted to our laboratory for Fragile X DNA testing. Of them 191 males belonging to the LCR group and of the 132 males belonging to the HCR group, 120 and 98 males were also tested for the loss of AGG interruptions. Written Informed consents were obtained from all the participants in this study.

### 2.2 Index of the patterns of loss of AGG

**Figure 1:**
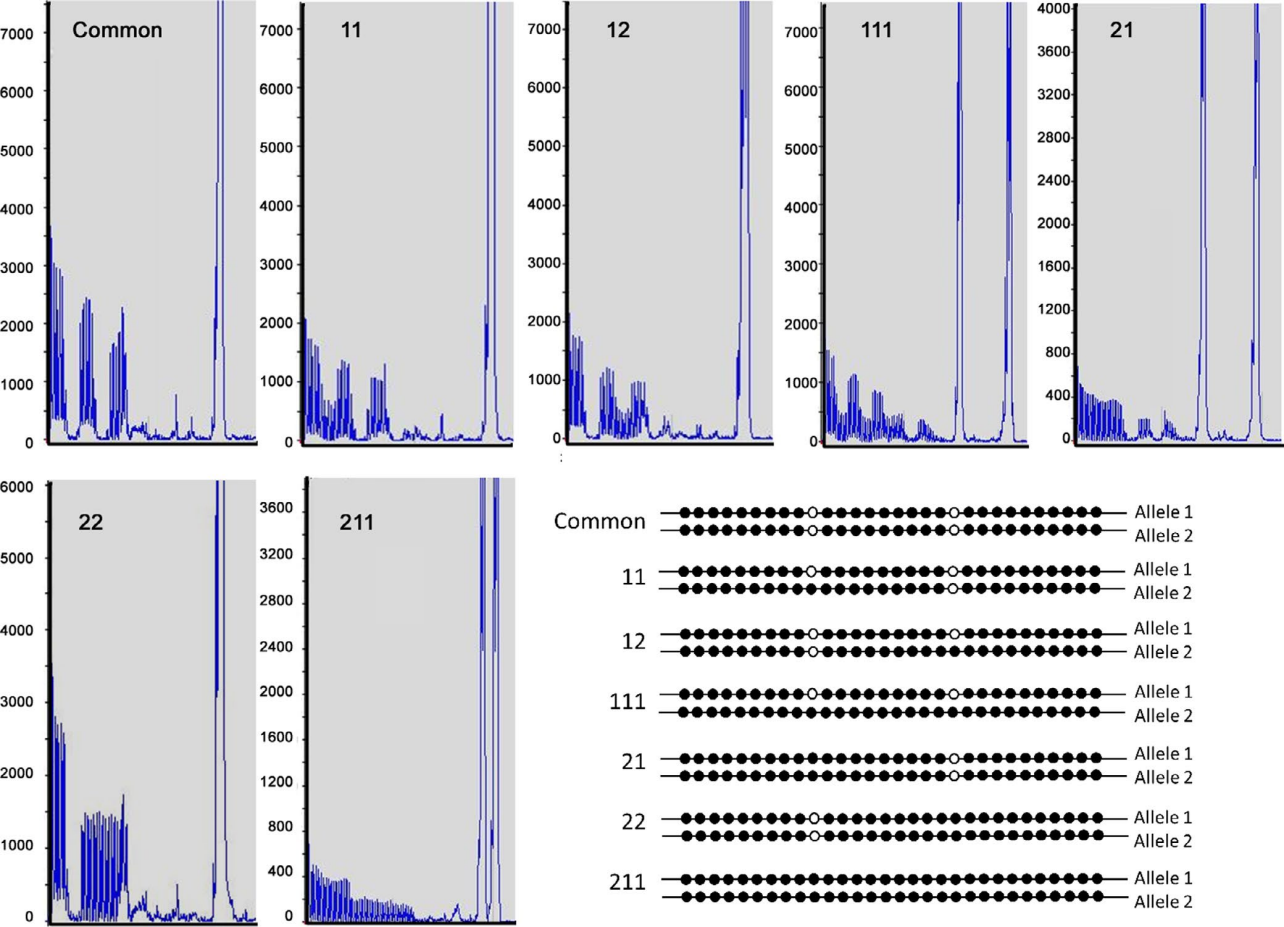
Index of AGG loss pattern

We defined the index of the patterns of loss of AGG (Figure 1) as follows:

11‐one AGG loss in the 1st position in one allele.

12‐one AGG loss in 2nd position in one allele.

111‐one AGG loss in 1st and 2nd position in one allele.

1122‐AGG loss in one allele in the 1st position and in both alleles in the 2nd position (not included in Figure 1,very rare cases).

21‐AGG loss in the 1st position in both alleles.

211‐AGG loss in the 1st position and 2nd position in both alleles.

22‐AGG loss in the 2nd position in both alleles.

2112‐AGG loss in both alleles in the 1st position and only in one allele in the 2nd position (not included in Figure 1,very rare cases).

### 2.3 Isolation of genomic DNA

Genomic DNA was isolated from peripheral blood lymphocytes using MagNa Pure LC DNA Isolation Kit (Roche applied Science) or QIAsymphony DNA Midi Kit (96) ‐931255 and the MagNa Pure or QIAsymphony machine according to the manufacturer's instructions.

### 2.4 Triple‐primed‐PCR

Genomic DNA (40–60 nanograms) was amplified with the Amplidex *FMR1* PCR assay (Asuragen, Austin TX) as previously described (Filipovic‐Sadic et al., 2010; Nahhas et al., 2012) and according to the manufacturer's instructions. Samples were analyzed by the 3130*xl* Genetic Analyzer (Applied Biosystems Inc.) and electropherograms were analyzed using GeneMapper 4.0 (4.1 for 3500xL data) (Nahhas et al., 2012) to determine CGG repeats length and the distribution pattern of the AGG interruptions. The accuracy of the CGG repeat number and AGG (presence/absence) were determined as ± 1 repeat and ± 0, respectively.

### 2.5 Haplotype analysis

In order to investigate if specific haplotypes may characterize the ethnic groups and pointing to a stability factor, we analyzed the following polymorphic makers: DXS548, FRAXAC1, rs25714 (IVS10), rs4949 (ATL1) located proximally and distally to the CGG repeats region of the *FMR1* gene. Two of them, DXS548 and FRAXAC1 were microsatellite markers and were genotyped and visualized using capillary gel electrophoresis. In addition, the two SNPs downstream of the CGG repeat element (rs25714 and rs4949) were analyzed using Taqman SNP genotyping following the manufacturer's protocols. Detailed method described by (Yrigollen, Mendoza‐Morales, Hagerman, and Tassone (2013)).

### 2.6 Statistical analysis

Statistical analysis was performed using SPSS. Chi‐squared test was used to assess the association between consanguinity and the loss of AGG sequences. Z‐ Score Calculations for 2 Population Proportions were used in order to determine whether the two groups differed significantly on some single (categorical) characteristic. *p*‐values less than .05 were considered statistically significant. *p*_z_ was defined in this study as the probability of *Z*‐test and *p*_chi_ as the probability of the Chi‐squared test. Correlations between the length of CGG repeats and the AGG loss for the different CGG categories were determined by Pearson correlation and by Spearman's Rho. *r*‐value of 1 by both correlation tests was considered as a positive perfect correlation and *p*‐values less than .05 were considered statistically significant.

## 3 RESULTS

### 3.1 Prevalence of CGG repeat allele size in the HRC and LCR groups

**Figure 2:**
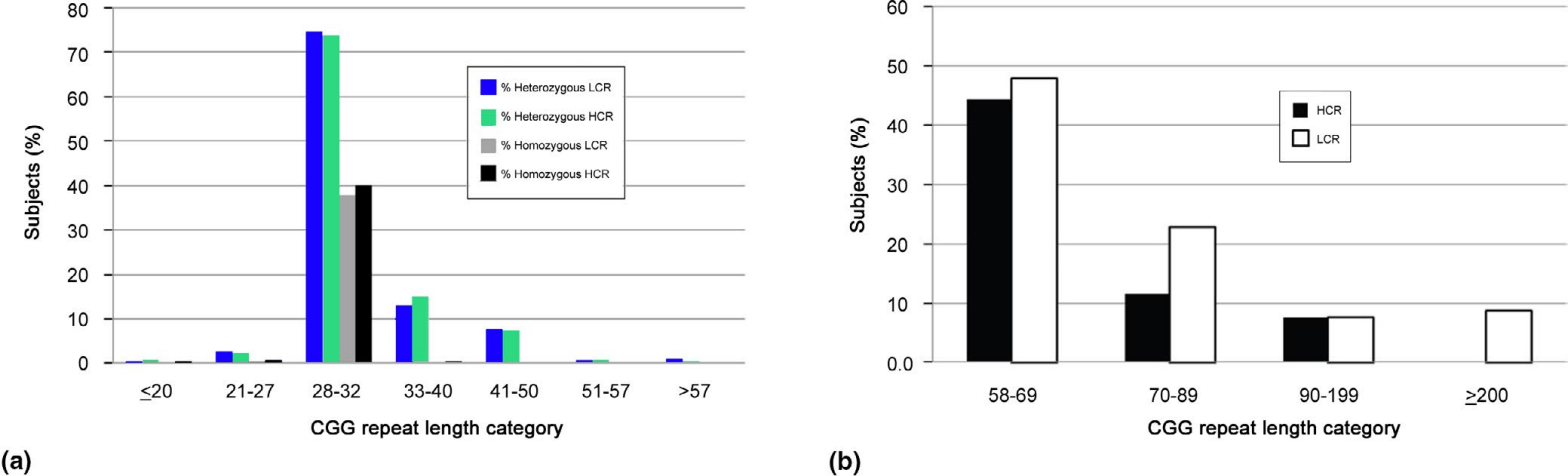
Prevalence of CGG repeats length in HCR and in LCR groups by percentages. A total of 17,087 samples were tested for CGG repeat length of which 7,893 were HCR subjects and 9,194 were LCR subjects. (a) Prevalence of subjects at ≤ 20÷57 CGG repeats categories. (b) Prevalence of subjects in the premutation and in the full mutation categories

A total of 17,087 females participated in this study; among them, 7,893 were from the HCR population and 9,194 belonged to the LCR population. In the HCR population: 7,843 (99.4%) females were in the ≤ 57 CGG repeat length category and 50 (0.6%) in the 58–199 CGG repeat premutation category. Most of the females (*n* = 35) belonged to the 58–69 CGG repeat category, 9 females to the 70–89 CGG repeat category and only 6 females carried an allele greater than 90 CGG repeats. The prevalence of the premutation in this population was 1 in 158. None of them had the full mutation. The LCR population included 9,114 females and 99.1% of them were in the ≤ 57 CGG repeat length category and 72 females (0.78%) in the 58–199 CGG repeat category. The prevalence of the premutation in the LCR population was 1 in 128. Eight females had the full mutation (>200 CGG repeats) and hence, the full mutation prevalence was 1 in 1,149. The highest CGG repeat allele length prevalence in both populations was in the 28–32 CGG range (Table 1 and Figure 2). Table 1 shows the proportion (number of subjects and respective percentages within the HCR and LCR groups) between the homozygous (0 or ± one CGG repeat difference between the alleles) and heterozygous (according to the higher CGG repeat allele). Figure 2a depicts the prevalence of both groups (HRC and LCR) within different CGG repeat ranges. Furthermore, we observed a higher prevalence of premutation and full mutation alleles in the LCR compared to the HCR group (Figure 2b).

**Table 1:** Prevalence of CGG repeat length categories in HRC and LRC ethnic groups in homozygous and heterozygous status

CGG repeats length categories	% Homozygous		% Heterozygous	
	HCR	LCR	HCR	LCR
≤20	44 (0.56)	26 (0.28)	51 (0.65)	41 (0.45)
21–27	55 (0.70)	53 (0.58)	184 (2.33)	256 (2.78)
28–32	3,170 (40.16)	3,480 (37.85)	5,813 (73.65)	6,867 (74.69)
33–40	44 (0.56)	20 (0.22)	1,168 (14.80)	1,195 (13.00)
41–50	15 (0.19)	14 (0.15)	561 (7.11)	699 (7.60)
51–57	2 (0.03)	0 (0.00)	66 (0.84)	56 (0.61)
>57	0 (0.00)	0 (0.00)	50(0.6)	80(0.9)
58–69	0 (0.00)	0 (0.00)	35 (0.44)	44 (0.48)
70–89	0 (0.00)	0 (0.00)	9 (0.11)	21 (0.23)
90–199	0 (0.00)	0 (0.00)	6 (0.08)	7 (0.08)
≥200	0 (0.00)	0 (0.00)	0 (0.00)	8 (0.09)
Total	3,330 (42)	3,593 (39)	7,893	9,194

Note

Percentage is indicated in parenthesis. Subjects were considered homozygous if the CGG difference between the two alleles was ± 1 CGG and heterozygous if the CGG difference between the two alleles was > 1 CGG repeat.

### 3.2 Homozygous and heterozygous patterns of AGG loss in the HRC and LCR groups

A total of 12,769 females: 6,204 females belonged to the HCR and 6,565 belonged to the LCR group, were also tested for the presence and distribution pattern of the AGG interruptions. Subjects were divided in groups according to their AGG pattern loss as defined in Materials and Methods (Figure 1). Table 2 shows the observed patterns of AGG loss in homozygous subcategory (the same CGG repeat length or 1 CGG repeat difference between the alleles) in both the HCR and LCR groups. Table 2 also shows that: (a) in both groups, the 28–32 CGG repeat allele length range was the most prevalent (38.1% in the HCR and 35% in LCR groups); (b) a statistically significant higher rate of homozygosity was observed in the HCR compared to the LCR group in the total CGG length repeat analyzed cases in each ethnic group (40.28% vs. 36.28% *p*_chi_ = .00001, *p*_z_ = .00544, also Table 3 sub‐section D); (c) a statistically significant higher rate of homozygosity(patterns 21, 211, 22) was observed in the HCR compared to the LCR group in the total AGG loss analyzed cases in each ethnic group (14% vs. 9.13%, *p*_chi_ = .000231, *p*_z_ < .05, Table 3 sub‐section E). No homozygosity was observed in the premutation/full mutation CGG repeat length range.

**Table 2:** AGG loss patterns in each CGG repeat length category in HCR and LCR groups dividing into two subgroups: homozygous and heterozygous

HCR−6204	AGG loss patterns (%) in homozygous sub category										
CGG repeat length categories	11	111	12	21	211	22	1,122	2,112	Total cases with AGG loss (%)	Number of cases with no AGG loss (%)	Total cases analyzed for CGG length repeats (%)
≤20	0 (0.00)	0 (0.00)	0 (0.00)	2 (0.03)	0 (0.00)	0 (0.00)	0 (0.00)	0 (0.00)	2 (0.03)	38 (0.61)	40 (0.64)
21–27	6 (0.10)	1 (0.02)	0 (0.00)	3 (0.05)	4 (0.06)	1 (0.02)	0 (0.00)	0 (0.00)	15 (0.24)	34 (0.55)	49 (0.79)
28–32	227 (3.66)	105 (1.69)	60 (0.97)	27 (0.44)	8 (0.13)	2 (0.03)	0 (0.00)	3 (0.05)	432 (6.96)	1932 (31.14)	2,364 (38.10)
33–40	2 (0.03)	3 (0.05)	1 (0.02)	19 (0.31)	3 (0.05)	1 (0.02)	0 (0.00)	1 (0.02)	30 (0.48)	7 (0.11)	37 (0.60)
41–50	0 (0.00)	0 (0.00)	0 (0.00)	5 (0.08)	1 (0.02)	1 (0.02)	0 (0.00)	1 (0.02)	8 (0.13)	0 (0.00)	8 (0.13)
51–57	0 (0.00)	0 (0.00)	0 (0.00)	0 (0.00)	1 (0.02)	0 (0.00)	0 (0.00)	0 (0.00)	1 (0.02)	0 (0.00)	1 (0.02)
Total	235	109	61	46	17	5	0	5	488 (19.53)	2011 (80.47)	2,499 (40.28)
Total homozygosity in the AGG loss category: 68 (14)											

LCR−6565											
CGG repeat length categories	11	111	12	21	211	22	1,122	2,112	Total cases with AGG loss (%)	Number of cases with no AGG loss (%)	Total cases analyzed for CGG length repeats (%)
≤20	0 (0.00)	0 (0.00)	0 (0.00)	0 (0.00)	0 (0.00)	0 (0.00)	0 (0.00)	0 (0.00)	0 (0.00)	16 (0.24)	16 (0.24)
21–27	7 (0.11)	2 (0.03)	0 (0.00)	3 (0.05)	0 (0.00)	3 (0.05)	0 (0.00)	0 (0.00)	15 (0.23)	22 (0.34)	37 (0.56)
28–32	219 (3.34)	56 (0.85)	64 (0.97)	23 (0.35)	1 (0.02)	5 (0.08)	1 (0.02)	1 (0.02)	370 (5.64)	1931 (29.41)	2,301 (35.05)
33–40	2 (0.03)	4 (0.06)	0 (0.00)	1 (0.02)	1 (0.02)	0 (0.00)	0 (0.00)	0 (0.00)	8 (0.12)	5 (0.08)	13 (0.20)
41–50	1 (0.02)	0 (0.00)	0 (0.00)	0 (0.00)	0 (0.00)	0 (0.00)	0 (0.00)	0 (0.00)	1 (0.02)	4 (0.06)	5 (0.08)
51–57	0 (0.00)	0 (0.00)	0 (0.00)	0 (0.00)	0 (0.00)	0 (0.00)	0 (0.00)	0 (0.00)	0 (0.00)	0 (0.00)	0 (0.00)
Total	229	62	64	26	2	8	1	1	394 (17)	1978 (83)	2,382 (36.28)
Total homozygosity in the AGG loss category: 36 (9.13)											

HCR−6204	AGG losses patterns (%) in heterozygous sub category										
CGG repeat length categories	11	111	12	21	211	22	1,122	2,112	Total cases with AGG loss (%)	Number of cases with no AGG loss (%)	Total cases analyzed for CGG length repeats (%)
≤20	1 (0.02)	0 (0.00)	0 (0.00)	3 (0.05)	0 (0.00)	0 (0.00)	0 (0.00)	0 (0.00)	4 (0.06)	43 (0.69)	47 (0.76)
21–27	32 (0.52)	21 (0.34)	0 (0.00)	5 (0.08)	4 (0.06)	1 (0.02)	2 (0.03)	3 (0.05)	68 (1.10)	82 (1.32)	150 (2.42)
28–32	525 (8.46)	382 (6.16)	154 (2.48)	66 (1.06)	20 (0.32)	5 (0.08)	1 (0.02)	10 (0.16)	1,163 (18.75)	3,403 (54.85)	4,566 (73.60)
33–40	236 (3.80)	305 (4.92)	19 (0.31)	63 (1.02)	24 (0.39)	5 (0.08)	0 (0.00)	20 (0.32)	672 (10.83)	259 (4.17)	931 (15.01)
41–50	87 (1.40)	182 (2.93)	8 (0.13)	32 (0.52)	21 (0.34)	2 (0.03)	2 (0.03)	12 (0.19)	346 (5.58)	67 (1.08)	413 (6.66)
51–57	3 (0.05)	35 (0.56)	0 (0.00)	3 (0.05)	5 (0.08)	0 (0.00)	0 (0.00)	8 (0.13)	54 (0.87)	3 (0.05)	57 (0.92)
58–69	1 (0.02)	19 (0.31)	0 (0.00)	1 (0.02)	3 (0.05)	0 (0.00)	4 (0.06)	0 (0.00)	28 (0.45)		28 (0.45)
70–89	1 (0.02)	6 (0.10)	0 (0.00)	0 (0.00)	1 (0.02)	0 (0.00)	0 (0.00)	0 (0.00)	8 (0.13)		8 (0.13)
90–199	0 (0.00)	3 (0.05)	0 (0.00)	1 (0.02)	0 (0.00)	0 (0.00)	0 (0.00)	0 (0.00)	4 (0.06)		4 (0.06)
≥200	0 (0.00)	0 (0.00)	0 (0.00)	0 (0.00)	0 (0.00)	0 (0.00)	0 (0.00)	0 (0.00)	0 (0.00)		0 (0.00)
	886	953	181	174	78	13	9	53	2,347 (37.83)	3,857	6,204
Total (%)	2013 (85.8)			265 (11.3)							

LCR−6565											
CGG repeat length categories	11	111	12	21	211	22	1,122	2,112	Total cases with AGG loss (%)	Number of cases with no AGG loss (%)	Total cases analyzed for CGG length repeats (%)
≤20	4 (0.06)	0 (0.00)	0 (0.00)	1 (0.02)	0 (0.00)	0 (0.00)	0 (0.00)	0 (0.00)	5 (0.08)	21 (0.32)	26 (0.40)
21–27	36 (0.55)	17 (0.26)	7 (0.11)	9 (0.14)	0 (0.00)	4 (0.06)	0 (0.00)	1 (0.02)	74 (1.13)	123 (1.87)	197 (3.00)
28–32	530 (8.07)	298 (4.54)	282 (4.30)	53 (0.81)	17 (0.26)	10 (0.15)	2 (0.03)	9 (0.14)	1,201 (18.29)	3,698 (56.33)	4,899 (74.62)
33–40	159 (2.42)	258 (3.93)	29 (0.44)	25 (0.38)	18 (0.27)	3 (0.05)	2 (0.03)	10 (0.15)	504 (7.68)	365 (5.56)	869 (13.24)
41–50	71 (1.08)	171 (2.60)	9 (0.14)	20 (0.30)	13 (0.20)	1 (0.02)	1 (0.02)	4 (0.06)	290 (4.42)	186 (2.83)	476 (7.25)
51–57	0 (0.00)	31 (0.47)	0 (0.00)	1 (0.02)	3 (0.05)	0 (0.00)	1 (0.02)	3 (0.05)	39 (0.59)	5 (0.08)	44 (0.67)
58–69	1 (0.02)	25 (0.38)	0 (0.00)	0 (0.00)	4 (0.06)	0 (0.00)	0 (0.00)	3 (0.05)	33 (0.50)		33 (0.50)
70–89	0 (0.00)	12 (0.18)	0 (0.00)	0 (0.00)	0 (0.00)	0 (0.00)	0 (0.00)	1 (0.02)	13(0.20)		13 (0.20)
90–199	6 (0.09)	0 (0.00)	0 (0.00)	0 (0.00)	0 (0.00)	0 (0.00)	0 (0.00)	0 (0.00)	6(0.09)		6 (0.09)
≥200	0 (0.00)	0 (0.00)	0 (0.00)	0 (0.00)	1 (0.02)	0 (0.00)	0 (0.00)	1 (0.02)	2(0.03)		2 (0.03)
	807	812	327	109	56	18	6	32	2,167(33)	4,398	6,565
Total (%)	1946 (89.8)			183 (8.4)							

Note

Distribution pattern of AGG loss in the two subgroups: homozygous (one CGG repeat difference between the two alleles or same CGG repeat length) and heterozygous. In parenthesis is the percentage of number of cases in each ethnic group (HCR, *n* = 6,204 and LCR, *n* = 6,565).

**Table 3:** Statistical test results for AGG loss for the different CGG repeat categories in the HCR group compared to the LCR group

	Patterns of AGG	Consanguinity		*p*‐value of Chi‐squared test *p*‐value of *z*‐test
		HCR *n* (%)	LCR *n* (%)	
A	All patterns of AGG loss at all CGG repeats categories	2,347 (37.8)	2,167 (33.0)	*p* = .00001 *p* = .001
B	All patterns of AGG loss at category ≤ 57 CGG repeats	2,307 (37.2)	2,113 (32.2)	*p* = .00001 *p* = .001
C	All patterns of AGG loss at category 58–199 CGG repeats	40 (0.6)	52 (0.8)	NS
D	Homozygous subjects (with no AGG losses)^a^ Heterozygous subjects (with no AGG losses)	2011 (32.4) 1846 (29.8)	1978 (30.1) 2,420 (36.9)	*p* < .00001 *p* = .00544
E	AGG pattern loss in two alleles: 211 + 21+22. Homozygous^a^ at CGG repeats and AGG losses No AGG loss (Homozygous at CGG repeats)	78 (1.3) 2011 (32.4)	37 (0.6) 1978 (30.1)	*p* = .000231 *p* < .05
F	Homozygous subjects (with no AGG losses)^a^ Heterozygous subjects (with no AGG losses)	3,330 (42) 4,563 (58)	3,593 (39) 5,601 (61)	*p* < .000037 *p* < .05

Note

The subject's percentages of AGG presence or loss for the different pattern of AGG loss is shown in parenthesis.

Abbreviation: NS, not significant.

a Subjects were considered homozygous if the CGG repeats difference between the two alleles was ± 1 and if the presence or loss of AGG were same (±0) at both alleles.

All patterns of AGG loss in the heterozygous subcategories (according to the higher CGG repeat allele length) in the HCR and LCR groups show that: (a) in both groups 28–32 CGG repeat length is the highest prevalent (73.6% vs. 74.6% in the HCR and LCR, respectively); (b) a statistically significant higher rate of AGG loss (for all patterns of AGG loss) was observed in the HCR compared to the LCR group in the total CGG length repeat (37.8% vs. 33% *p*_chi_ = .00001, *p*_z_ < .001, Table 3 sub‐sections A and B); (c) higher rate of AGG loss patterns in one allele: 11,111,12 were observed in the LCR compared to the HCR group (89.8% vs. 85.8%) and higher rate of AGG loss pattern in both alleles: 2,121,122 in the HCR compared to LCR (11.3% vs. 8.4% *p*_chi_ = .000231).

### 3.3 Statistical analysis

The statistical analysis, summarized in Table 3, shows that statistically significant differences between the two ethnic groups, were found for the following parameters: loss of AGG, homozygosity, and patterns of AGG loss in both alleles.

### 3.4 The absence of AGG increases with increased CGG repeat number

**Figure 3:**
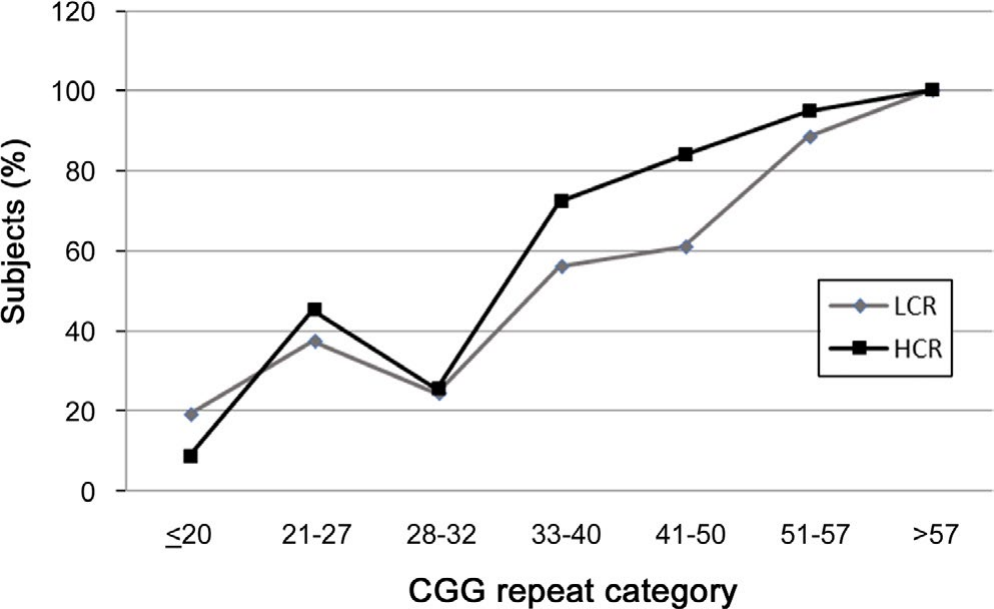
Correlation between AGG loss and the CGG repeat length. A linear correlation between the overall AGG loss patterns increased with the increased CGG repeat number

A linear correlation between the overall AGG loss which increases as the CGG repeat increases is shown in Table 4 and in Figure 3. Both ethnic groups show the same correlation between the CGG repeat length and loss of AGG. Our results show that, in general, a positive correlation exists between CGG length and AGG loss in both HCR and LCR populations (homozygous and heterozygous). In addition, positive correlations (r = 1 and *p* < .05) were obtained for both HCR and LCR‐heterozygous subjects indicating that their statistic distributions are nonparametric.

**Table 4:** All AGG loss patterns in each CGG repeat length category in the HCR and LCR groups

CGG repeat length categories	Number of subjects in each CGG repeat length category in the two ethnic groups		All patterns of AGG loss (%)	
	HCR	LCR	HCR	LCR
≤20	47	26	4 (8.5)	5 (19.2)
21–27	150	197	68 (45)	74 (37.5)
28–32	4,566	4,899	1,163 (25.5)	1,201 (24.5)
33–40	931	869	672 (72.2)	504 (56.25)
41–50	413	476	346 (83.8)	290 (61)
51–57	57	44	54 (94.7)	39 (88.6)
>57	40	54	40 (100)	54 (100)

Note

Percent of AGG loss and CGG repeat length in the HCR and LCR ethnic group. In parenthesis is the percentage of the overall AGG loss in each CGG repeat length category.

### 3.5 Pattern of AGG loss profiles in the two ethnic groups

**Figure 4:**
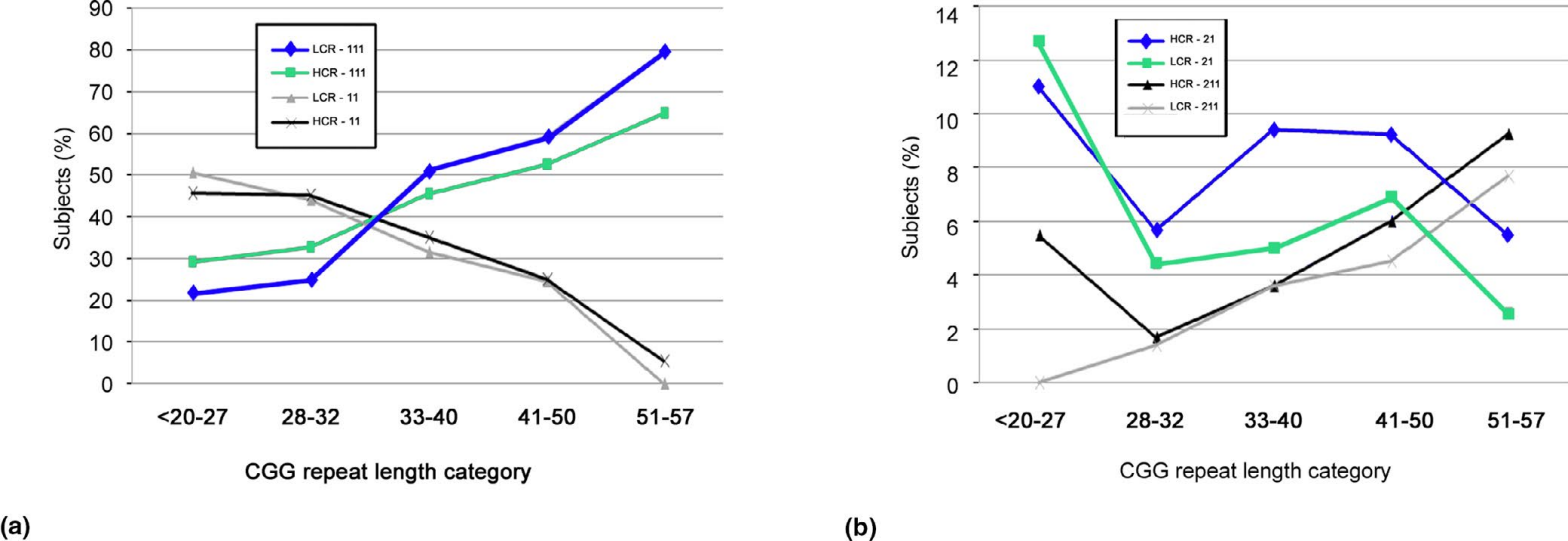
The tendencies of 11 and 111 (a) and 21 and 211 (b) AGG loss patterns in HCR and LCR groups. No statistical significant difference between the profile of AGG loss pattern in one allele of the ethnic groups was found, while a statistical significant difference was observed between the two ethnic groups in the profile of AGG loss pattern of two alleles (*p* = .000231; *p* < .05)

The different patterns of AGG loss in one allele (11,111,12) and in two alleles (21, 211,22) in each ethnic group are shown in Table 5 (a and b) and in Figure 4 (a and b). Table 5a and Figure 4a shows that the AGG loss pattern 11 is more prevalent then AGG pattern 111 but only up to 28–32 CGG repeat length, while beyond 32 CGG repeats, the pattern of AGG loss 111 becomes more prevalent. No statistical differences were observed between the two ethnic groups. However, the prevalence of the overall AGG loss was statistically significant higher in the HCR compared to LCR group. (*p*_chi_ = .000039, *p*_z_ < .05).

**Table 5:** Pattern of AGG loss in one allele (a) and in two alleles (b) in the HCR and LCR groups. a) The correlation between the percentage of pattern of AGG loss (11,111,21) and b) (21,211.22) calculated according to the total number of cases with AGG loss in the relevant CGG repeat length category

(a)								
CGG repeat length categories	AGG loss patterns in one allele (%)						Total cases with AGG loss	
	11		111		12			
	HCR	LCR	HCR	LCR	HCR	LCR	HCR	LCR
<20–27	33 (45.8)	7 (9.1)	0 (0.00)	17 (21.5)	21 (29.1)	40 (50.6)	72	79
28–32	525 (45.2)	282 (23.48)	154 (13.24)	298 (24.8)	382 (32.8)	530 (44.12)	1,163	1,201
33–40	236 (35.1)	29 (5.75)	19 (2.8)	258 (51.2)	305 (45.4)	159 (31.54)	672	504
41–50	87 (25.15)	9 (3.1)	8 (2.3)	171 (59)	182 (52.6)	71 (24.5)	346	290
51–57	3 (5.5)	0 (0.00)	0 (0.00)	31 (79.5)	35 (64.8)	0 (0.00)	54	39

(b)								
CGG repeat length categories	AGG loss patterns in two allele (%)						Total cases with AGG loss	
	21		211		22			
	HCR	LCR	HCR	LCR	HCR	LCR	HCR	LCR
<20–27	8 (11)	10 (12.65)	4 (5.5)	0	1 (1.4)	4 (5)	72	79
28–32	66 (5.67)	53 (4.4)	20 (1.7)	17 (1.4)	5 (0.4)	10 (0.83)	1,163	1,201
33–40	63 (9.4)	25 (5)	24 (3.6)	18 (3.57)	5 (0.74)	3 (0.6)	672	504
41–50	32 (9.24)	20 (6.9)	21 (6)	13 (4.5)	2 (0.6)	1 (0.34)	346	290
51–57	3 (5.5)	1 (2.56)	5 (9.25)	3 (7.7)	0 (0.00)	0 (0.00)	54	39

The patterns of AGG loss in both alleles revealed that in general, their prevalence in both ethnic groups was low: 11.2% versus 8.4% in HCR and LCR group. Since the number of subjects in each category (*n* = 265 and *n* = 183) was low we could not show statistical significance, yet, significant difference was observed in the pattern of AGG loss 21 in the HCR compared to LCR group in the 33–40 CGG repeat range (*p*_chi_ < .00001, *p*_z_ < .05).

### 3.6 FXS analysis in males

Loss of AGG interruptions was assessed in 323 males. Of them, 191 males belonged to LCR group; 120 were tested also for AGG loss. We found that fifteen percent of them had the 11 and 111 pattern of AGG loss. Six males had an allele in the premutation range and six males had the full mutation (>200 CGG repeats). Within the 132 males belonging to the HCR group two had a premutation allele (60 and 67 CGG repeats). The AGG loss, determined for 98 males, was almost twofold higher in the HCR than in the LCR group (29% vs. 15%).

### 3.7 Mosaicism in the normal range

During our routine testing for FXS that occurred between 2016 and 2017, we found 16 cases of 5,994, with alleles of three different CGG repeat sizes and eight cases showed AGG loss. Specifically, the 111 patterns of AGG loss were observed in three cases,while, the 11 patterns of AGG loss were observed in five cases.

### 3.8 Haplotype analysis

No significant differences were observed between the HCR and LCR groups in the haplotype analysis performed on 147 and 144 samples from LCR and HCR groups, respectively. However the level of homozygosity was significantly higher (*p*_z_ < .0001 in the HCR compared to the LCR group.

## 4 DISCUSSION

The AGG interruptions within the CGG repeat region of the *FMR1* gene, which usually occur after every 9 or 10 CGG triplets (Yrigollen et al., 2014), are well known as an important element for the stability of the CGG repeat length within the *FMR1* gene (Eichler et al., 1994; Ennis, Murray, Brightwell, Morton, & Jacobs, 2007; Nolin et al., 2015, 2013; Yrigollen et al., 2012, 2014; Zlotogora, Grotto, Kaliner, & Gamzu, 2015). However, some evidences raise the question regarding the strength of this theory. One of them is the instability found within the normal range of the CGG repeats length. We and others (Sharony et al., 2012; Wakeling et al., 2014) have indeed reported instability of alleles within the normal range (< 55 CGG repeats). Sharony et al. (2012) and Wakeling et al. (2014) reported on the presence of an extra allele with a prevalence of ~0.07% and 0.4%, respectiveley, in the general population. In our routine *FMR1* screening testing of the general population (between 2016 and 2017), we observed the presence of an extra allele within the normal range in 0.27% of the cases (16 of 5,994 Females). An AGG loss was found only in half (*n* = 8) of our cases. Another question regards the role of AGG on the stability of the *FMR1* allele which was reported to be limited only up to approximately 90 CGG repeats, while, beyond this point no stabilization effect is observed (Nolin et al., 2015; Yrigollen et al., 2014). Additionally, expanded alleles are almost exclusively transmitted by females in following generations. Males usually transmit alleles they do not seem to expand to full mutation allele regardless the presence or absence of AGGs or the CGG repeat length.

Inter marriage (consanguinity) within families decreases the genomic variability and increases the homozygosity rate. In our study, we looked at the role of AGG in the instability of the *FMR1* CGG repeat by comparing two ethnic populations that differed mainly in their consanguinity rate (~45%–70% vs. ~5%). We expected an increase rate of AGG loss that according to the AGG stability theory should have increased the prevalence of the premutation/full mutation prevalence. However, in the HCR population, the prevalence of the premutation was 1 in 158 and no full mutation was detected. Specifically, 0.6% females carried an allele in the premutation range (58–199 CGG repeat) in 12% greater than 90 CGG repeats. In comparison the prevalence of the premutation in the LCR population was 1 in 128:0.9% of the females carried an allele > 57 CGG repeats and among them 11.5% were above 200 CGG repeats, with a the full mutation prevalence of 1 in 1,149.

The premutation prevalence in Israel according to the Ministry of Health as described by Zlotogora et al. (2015) for 44,592 tested women was 1:149, for the Jews 1:121 and for the Muslin Arabs 1:264. According to Berkenstadt, Ries‐Levavi, Cuckle, Peleg, and Barkai (2007) and Toledano‐Alhadef et al. (2001) the Jewish cohort showed a prevalence of 1:157 and 1:113 respectively when the carrier range was greater than 54 CGG repeats. The prevalence of the premutation is different in different regions of the world; in United State it is 1:178–430 (Hantash et al., 2011; Maenner et al., 2013; Tassone et al., 2012) whereas in Quebec (Rousseau, Rouillard, Morel, Khandjian, & Morgan, 1995) it is 1:259–1:397 while in the far east it is significantly lower (Otsuka et al., 2010; Tzeng et al., 2005). No full mutation was found in the HCR population over a 6 years study period while in the LCR population the incidence of the full mutation was 1 in 1,149 females (Table 1 Figure 2b). This represents a high prevalence of a full mutation compared to the worldwide rate, except to the one observed in an area of Colombia, likely due to a founder effect (Saldarriaga et al., 2018). From a review by Peprah (2012), the worldwide prevalence of the syndrome varies from 1:2,359 in Spain to 1:27,115 in Estonia. In Israel, Toledano‐Alhadef et al. (2001) reported a prevalence of 1 in 4,778 and Weiss et al. (2014) reported a prevalence of 1 in 3,867. It is important to note that in this study, the prevalence of the full mutation in females was obtained through genetic testing, regardless the phenotypic expression. This might be reason for the difference between our findings and those from others. Moreover, no full mutation was found among the 132 HCR males and only two carried a premutation allele while six males with the full mutation and six males with an allele in premutation range were identified among 191 LCR males.

This is the first study describing consanguinity as related to the premutation prevalence in Israel. Compared to the different published prevalence it is the lower prevalence found in the HCR population in the Negev Region in Israel.

The prevalence of all patterns of AGG loss observed was approximately 35%, which is much higher than that published by Weiss et al. (2014). In their study, the number of individuals was much lower than in our study (624 vs. 12,769) and included 326 Ashkenazi and 298 non‐Ashkenazi women. They found that only 9% of the Ashkenazi group lost AGG as compared to 19% of the non‐Ashkenazi group. To the best of our knowledge, no such big cohort as the one presented here, studying the patterns of AGG loss within the normal population has been previously reported.

Our results showed that in general the loss of AGG is highly prevalent in both populations within the normal CGG repeat range, 37.2% versus 32.2% in the HRC and LRC populations, respectively. The results showed a significant association between consanguinity and AGG loss. This association was significant for all patterns of AGG loss observed within the entire CGG repeat range (*p*_chi_ = .00001, *p*_z_ = .001) (Table 3).

This is the first report showing that the loss of AGG is highly prevalent in the normal population. These results indicate that the loss of AGG may also be a polymorphic event. Most published data concentrated on the loss of AGGs within the premutation and full mutation CGG repeat range (Nolin et al., 2003, 2015, 2013; Yrigollen et al., 2012, 2014). Thus, the lack of studies looking at the prevalence of AGG loss within the normal CGG length range may have misled us regarding the importance of AGG role in the *FMR1* stability.

The most prevalent CGG repeat length allele in both ethnic groups (Tables 1 and 2, Figure 2) was in the range of 28–32 CGG (Table 1 and Figure 2), in both the homozygous (the same CGG length or one repeat different between the two alleles) and in the heterozygous status, which is in agreement with other published data (Peprah, 2012; Tassone et al., 2012; Weiss et al., 2014).

We observed for the first time that there is a specific profile of AGG loss pattern related to the CGG repeat length categories with no statistical significant difference between the two ethnic groups. When looking at alleles with up to 32 CGG repeats the most prevalent pattern of AGG loss in both the HCR and LCR groups was 11 (59% and 55%, respectively), followed by the pattern 111 (24% and 15%, respectively) and then by pattern 12 (14% and 17%, respectively). However, this profile changed beyond 32 CGG repeat length, where, the most prevalent AGG pattern loss was 111 followed by pattern 11 and then by pattern 12 (Table 5a and Figure 4a). This might be explained by a possible progressive development in which one AGG loss happens in the low CGG repeat length range as first event, while, the loss in the second position may occur as a second event occurring with increased CGG repeat length. However, although is a very rare event, we could not explain how the loss of the AGG in the second position only (pattern 12) occurs.

Theoretically there might be two options for the presence of AGG interruptions every 9–10 CGG repeats within the *FMR1* gene: either the ancestral allele did not contain AGG interruptions and what we see now is a gain of AGG interruptions or alternatively the loss of AGG occurred from an ancestral gene containing AGG interruptions. Our study strengthens the second option as about 70% of each population have AGG interruptions.

The patterns of AGG loss 21, 211, and 22 are the result of mating of individuals carrying each one the same pattern of AGG loss. Indeed, we found a statistical significant association between these patterns of AGG loss and consanguinity (*p*_chi_ = .00001 and *p*_chi_ = .00209 respectively).

It appears that the loss of AGG in one allele occured in a universal mechanism regardless ethnicity while the loss of AGG in the second allele may have reflected the effect of consanguinity and homozygosity. However, the mechanism by which AGG loss occurred and its correlation with the increasing CGG repeats length is still unknown and needs further studies.

We found that the homozygosity, as well as, the rate of AGG loss were statistically significantly higher in the HCR as compared to the LCR population (*p*_chi_ = .00001, *p*_z_ = .00544).The loss of AGG was 4.8% higher in the HRC group compared to the LRC group. Although the association between consanguinity and homozygosity was expected, the low prevalence of the full mutation in the HCR population (no full mutation cases were detected in 7,854 females and 132 males) is not in agreement with the theory of the AGG loss as an important factor in the instability of the *FMR1* gene.

It can be concluded, from the data presented here, that AGG interruptions, particularly within the normal range are not necessarily only stabilizing the *FMR1* gene and that their presence or absence could be related to a polymorphism. We think that there may be other stabilization factors, likely ethnic distinct, which prevent, an high prevalence, of premutation and full mutation alleles. Interestingly, Latham, Coppinger, Hadd, and Nolin (2014) showed that within the 70–79 CGG repeat range, the risk for expansion is 54% in FXS families in compared to 11% in families without FXS. Also Falik‐Zaccai et al. (1997) showed high prevalence of premutation and full mutation in the Tunisian ethnicity among the Jewish population, likely related to unique founder effect and genetic drift phenomena for accumulation of predisposed alleles in the population. Limprasert et al. (2016) showed that specific haplotype were associated with the loss of AGG interruptions. Recently, Sun et al. (2018) showed that disease‐associated tandem repeats are located to TAD boundaries and affect their insulation. The findings have important implications for TAD function and mechanisms underlying diseases such as FXS and Huntington's disease.

In summary, as expected, our results demonstrate that consanguinity affects the homozygosity as well as the prevalence of AGG loss. However, it did not affect the prevalence of the premutation and full mutation of the *FMR1* gene in the HRC group. The study of Shawky, Elsayed, Zaki, El‐Din, and Kamal (2013) aimed to determine the effect of consanguineous marriage (54.4% of the Egyptian group studied) on different types of genetic diseases and showed that child morbidity and mortality did not have a significant effect on the prevalence of FXS (*p* < .001). Finally, Weiss et al. (2014) showed no correlation between the loss of AGG (lower rate) and the prevalence premutation/full mutation in the Ashkenazi Jews compared to the non‐Ashkenazim group (higher rate of AGG loss). Both studies strengthen our results, namely that AGG may not be the only factor playing a role in the stability of the *FMR1* gene.

According to our results it could be suggested that the loss of AGG is polymorphic phenomenon in the general population that play also a role in the stability of the CGG repeat length in the *FMR1* gene. Although we did not find difference in the haplotype analysis between the two groups, the involvement of an ethnic distinct stabilization factor could still play an important role. Our results also show that there might be a tendency in the pattern and rate of AGG loss positively correlated to the CGG repeat length.

Finally, further studies are warranted to clarify these results as well as the mechanism of *FMR1* instability, which is, to date, still not fully understood.

## CONFLICT OF INTEREST

There is no author in this paper with conflict of interest.
